# Identification, technological and safety characterization of *Lactobacillus sakei* and *Lactobacillus curvatus* isolated from Argentinean anchovies (*Engraulis anchoita*)

**DOI:** 10.1186/2193-1801-2-257

**Published:** 2013-06-07

**Authors:** Carolina Belfiore, Raúl R Raya, Graciela M Vignolo

**Affiliations:** Centro de Referencia para Lactobacilos (CERELA), CONICET, Tucumán, Argentina; Chacabuco 145, 4000 San Miguel de Tucumán, Argentina

**Keywords:** *L. sakei*, *L. curvatus*, Anchovy, Molecular identification, Safety and technological traits

## Abstract

In this study, the identification and characterization of *Lactobacillus* previously isolated from fresh anchovies (*Engraulis anchoita*) are investigated. 16S rDNA partial sequencing assigned all the isolates to belong to the *Lactobacillus sakei/curvatus* group. Fourteen out of 15 isolates were identified as *L. sakei* by phenotypic traits: they exhibited catalase activity and fermented melibiose, although only 10 of them hydrolyzed arginine. These results were confirmed by multiplex PCR-based restriction enzyme analysis with *Hin*dIII and by restriction fragment length polymorphic (RFLP) analysis of the 16S-23S rDNA intergenic spacer region with *Taq*I. Among identified isolates, four *L. sakei* strains and the sole *L. curvatus* strain showing sensitivity to chloramphenicol, erythromycin and tetracycline and exhibiting high tolerance to NaCl (10-18%) were unable to produce neither dextran nor biogenic amines. Based on technological and safety features, *L. sakei* SACB_7_04 and *L. curvatus* SACB03a naturally present in fresh anchovies may be promising strains for the development of a starter culture to accelerate and control the fermentation of salt fermented anchovy-based products.

## Introduction

Fish has been one of the main foods for humans for many centuries and still constitutes an important part of the diet in many countries. The advantages of fish as a food are its easy digestibility and high nutritional value. Since more than half earth’s surface is covered by water, there are plenty of fish sources; the range of fish products being very large including foods prepared using a broad spectrum of both traditional and modern food technologies. Anchovy (*Engraulis anchoita*) is one of the most abundant pelagic fish species in the Southwest Atlantic ocean, in the so-called “anchoíta Bonaerense” stock, with a biomass of around 4,300,000 tons/year and a maximum sustainable yield of 1,140,000 tons/yr (Cabrer et al. 2002), being at present underexploited at 30,000 tons/year (SAGPyA 2008). In order to obtain products with typical sensory characteristics different from those of fresh fish, alternatives for new product development, in particular fermented and marinated anchovies have been considered (Hasan and Halwart 2009).

Although the muscle of living fish is sterile, the skin, mucus, gills and gut contain significant bacteria, whose composition and quantity vary according to the fish species, temperature, salinity and dissolved oxygen of the water, degree of pollution, feed and stress among other (Gram and Huss 1996;Gram and Dalgaard 2002). Recently, a considerably diversity of lactic acid bacteria (LAB) has been reported to colonize live fish either regularly or transiently (Michel et al. 2007). Numerous studies confirmed LAB as part of the microbiota of healthy marine and freshwater fish and their surrounding environment (Seppola et al. 2006;Balcázar et al. 2007;Leroi 2010;Lauzon and Ringø 2012). Although a few LAB species have been described as fish pathogens (Ruiz-Zarzuela et al. 2005;Vendrell et al. 2006;Michel et al. 2007;Liu et al. 2009;Svanevik and Lunestad 2011), most indigenous LAB are harmless and are considered as potential functional cultures in the production of numerous fermented fish products or as probiotics that could improve fish health.

On the other hand, technological processes such as vacuum-packaging, drying, salting and marinating applied to fresh fish to extend shelf-life lead LAB to be the predominant microbiota (Lyhs et al. 2002;González-Rodríguez et al. 2002;Thapa et al. 2006;Najjari et al. 2008;Matamoros et al. 2009;Kopermsub and Yunchalard 2010). The presence and dominance of LAB is highly variable depending on fish species and aquatic environments, natural and aquacultural as well as processing conditions (Leroi 2010;Lauzon and Ringø 2012). In particular for anchovy, *Pediococcus halophilus* and *Lactobacillus sakei* were described as the predominant species of Argentinean salted anchovies (Villar et al. 1985) and Tunisian salted raw anchovies (Najjari et al. 2008), respectively. Recently, *Leuconostoc mesenteroides* and *Leuc. carnosum* were identified as the predominant LAB population in Argentinean anchovies from 2005–2006 catch season (Belfiore et al. 2010). Based on the available and abundant biomass of this small pelagic fish, the selection of competitive LAB strains should allow designing new fermented anchovy-based products. In this study, a polyphasic approach was used for the identification and technological characterization of *Lactobacillus* strains in view to select an appropriate starter culture to improve stability and fermentation duration of salted anchovies

## Material and methods

### Sampling and LAB isolation

Anchovies (*E. anchoita*) caught between 34 and 36° Atlantic South latitude were provided by a fish processing factory from Mar del Plata (Argentina) and conditioning as previously reported (Belfiore et al. 2010). Ten grams of fresh anchovies were transferred into sterile stomacher bags; 90 ml of saline-peptone water (bacteriological peptone, 0.1% and NaCl, 7.5%) was added and mixed for 2 min in a stomacher machine (Stomacher Lab-Blender 400, A.J. Seward Lab. London, England). Tenfold dilution series were then prepared and the following analyses were carried out: total counts on Plate Count Agar (PCA) incubated for 48 h at 30°C and LAB on Man Rogosa and Sharpe (MRS) (de Man et al., 1960), incubated for 5 days at 25°C. Plates were inoculated using the spread plate technique and ten colonies were randomly picked from each sampling. After characterization by cell morphology, Gram reaction (Gram staining kit, Britania) and catalase activity (H_2_O_2_ 3% v/v was added to a glass slide containing fresh colonies from Petri plates; bubbles indicated positive result), a total of 15 isolates were collected for further analysis (SACB03a, SACB_7_01, SACB_7_02, SACB_7_04, SACB_7_05, SACB_7_06, SACB_7_08, SACB_7_09, SACB_7_010, SACB_7_02a, SACB_7_05a, SACB_7_06a, SACB_7_07a, SACB_7_09a, SACB_7_010a). Selected isolates were purified on MRS plates and then kept at −70°C in MRS broth containing 30% (v/v) glycerol.

### DNA extraction/purification and 16S rDNA gene sequence analysis

Chromosomal DNA of the fifteen isolates was prepared as described by Pospiech and Neumann (1995). Universal primers PLB and MLB (Kullen et al. 2000), Bact 8 F (Edwards et al. 1989) and 1391R (Lane et al. 1985) were used to partially amplify the 16S rDNA gene by PCR (Table 1). Amplification products, purified from agarose gels with silica beads (Sambrook et al. 1989), were sequenced at Cornell University Life Sciences Laboratories Center (Cornell University, USA). The 16S rDNA sequences similarity was compared with the GenBank data library using the nucleotide BLAST program (http://www.ncbi.nlm.nih.gov). Sequences were aligned using ClustalW (http://workbench.sdsc.edu, San Diego Supercomputer Center, University of California, San Diego) and the phylogenetic analysis was done with the Ribosomal Database Project Release 10 (RDP) program (http://rdp.cme.msu.edu;Cole et al. 2008). The 16S rDNA sequences of *Lactobacillus curvatus* DSM20019 (T), *Lactobacillus sakei* subsp. *sakei* DSM20017 (T), *L. sakei* subsp. *carnosus* CCUG34545 (T) were used as reference strains while *Enterococcus casseliflavus* AF039903 (T) was used as outgroup strain. The 16S rDNA sequences of isolates were deposited in the GenBank database under the following accession numbers: GQ205421, GQ205422, GQ205423, GQ205424, GQ205425, GQ205427, GQ205428, GQ205429, GQ205430, GQ205431, GQ205432, JQ653151, GQ303170, JQ653150 and GQ303174.

**Table 1:** **PCR primers and amplification conditions**

Primer	Sequence 5’→3’	References	PCR conditions *			
			Denaturing	Annealing	Extension	Cycles
PLB	AGAGTTTGATCCTGGCTCAG	Kullen et al. (2000)				
MLB	GGCTGCTGGCACGTAGTTAG		94°C, 1 min	50°C, 1 min	72°C, 1 min	30
Bact 8 F	AGAGTTTGATCCTGGCTCAG	Edwards et al. (1989)				
1391R	GACGGGCGGTGTGTRCA	Lane et al. (1985)	94°C, 1 min	56°C, 1 min	72°C, 1 min	30
16S/p2	CTTGTACACACCGCCCGTC	Gürtler and Stanisich (1996)				
23S/p7	GGTACTTAGATGTTTCAGTTC		94°C, 1 min	56°C, 1 min	72°C, 1 min	35
CS-f	GAGCTTGCTCCTCATTGATAA	Lee et al. (2004)				
CS-r	TTGGATACCGTCACTACCTG					
Uni-f	GATAAACCAAATGTGTAGGG		94°C, 0.5 min	58°C, 0.5 min	72°C, 1 min	35
M13	GAG GGT GGC GGT TCT	Huey and Hall (1989)	94°C, 1 min	45°C, 0.2 min	72°C, 2 min	40
RAPD1	AGC AGG GTC G	Cocconcelli et al. (1995)	94°C, 1 min	29°C, 1 min	72°C, 2 min	20
RAPD2	AGC AGC GTG G		94°C, 0.5 min	55°C, 0.5 min	72°C, 0.3 min	45

*All PCR programs included a first step of 94°C, 5 min and a final extension at 72°C, 5 min.

### Phenotypic assays

For each isolate, melibiose and xylose fermentation was assessed inoculating 1% of an overnight culture into glucose-free MRS broth supplemented with 2% of melibiose or xylose and chlorine phenol red (0.0018%), while a MRS medium supplemented with arginine (3 g/L) was used for arginine hydrolysis evaluation (Berthier and Ehrlich, 1999). The presence of haem-dependent catalase activity was detected upon addition of 20 μL of H_2_O_2_ (10 vol) on isolates grown in MRS broth containing glucose (5 g/L) and haematin-porcine (30 μg/L; Sigma). In all of cases, cultures were incubated at 30°C for 48 h. *L. sakei* 23 K (Unité Flore Lactique, INRA, Jouy-en-Josas, France), *L. sakei* CRL978 (ATCC15521), *L. curvatus* CRL705 (CERELA Culture Collection), *L. curvatus* subsp. *curvatus* CRL1000 (DSM20019) and *Lactobacillus graminis* DSM20719 were included as reference strains.

### Multiplex PCR-based restriction enzyme analysis and RFLP analysis of the 16S-23S rDNA intergenic spacer region (ISR)

Polymerase chain reactions (PCR; see conditions in Table 1) were performed in a total volume of 50 μL containing 200 μM of each deoxyribonucleoside triphosphate, 10 ng genomic DNA of each studied strain, 1.5 mM MgCl_2_, buffer reaction (1×) and 1.25 U of *Taq* DNA polymerase (Invitrogen, Brazil). Reactions were carried out in a BioRad thermocycler and negative controls without DNA template were included. Multiplex PCR and restriction enzyme analysis (REA) were carried out as described by Lee et al. (2004) while the ISR PCR amplification was performed using primers 16S/p2 and 23S/p7 as reported by Gürtler and Stanisich (1996). Restriction enzymes, *Hin*dIII for REA and *Taq*I for RFLP analysis, were provided by Biodynamics (Argentina). Digestion reactions were performed in a final volume of 20 μL at the optimal temperature according to the manufacturer’s protocols, using an AccuBlock digital dry bath (Labnet). Digestion products were run at 85 V in a 2% (w/v) agarose gel (Biodynamycs, Buenos Aires, Argentina); stained with ethidium bromide and visualized with UV light. Gel images were digitized with a charge-coupled camera (Canon) and stored as JPG files for further analysis.

### Genotypic fingerprinting

Isolates were analyzed by Random Amplification of Polymorphic DNA (RAPD) PCR using primers RAPD1, RAPD2 (Cocconcelli et al. 1995) and M13 (Huey and Hall 1989) (Table 1). Each primer was singly used in RAPD reactions performed in a reaction volume of 50 μL containing 3 mM MgCl_2,_ buffer reaction (1×), deoxyribonucleoside triphosphate (200 μM each), 1 μM of each primer, DNA (10 ng) and *Taq* polymerase (0.5 units; Invitrogen, Brazil). RAPD products were electrophoresed at 85 V on 2.5% (w/v) agarose gel (Biodymanics, Buenos Aires, Argentina), stained with ethidium bromide and, after washing, photographed under UV illumination using a Canon (power shot G6) camera.

### Technological and safety characterization of *Lactobacillus*

Bacterial growth of all isolates was tested in a muscle extract obtained from macerated fresh anchovy diluted with distilled water (1:10 v/v) and homogenized in a Stomacher 400 blender (London, UK) for 3 min. After centrifugation at 14000 × g for 20 min at 4°C, glucose 1% (w/v) was added to the supernatant and finally filter sterilized through 0.22 μm membrane (Steritop® Filter Unit, Millipore). The obtained muscle extract was inoculated with 1% (v/v) of an overnight culture of each isolates and incubated at 30°C for 48 h. Dextran-producing colonies were assayed by streaking plate on McClesky-Fanville agar (McClesky et al. 1947) incubated at 30°C for 48 h. Mucoid colonies were considered as dextran (+) strain; *L. sakei* CRL1424 (CERELA Culture Collection) was used as indicator organism. Growth and survival of isolates in the presence of salt (NaCl) was assessed in the range of 10 to 18% (w/v) NaCl. Over-night culture of each strain was used to inoculate (1% v/v) 25 ml of muscle extract with different NaCl concentrations. Samples were taken periodically (during seven months) and viability in MRS agar (30°C for 48 h) was determined.

Safety traits of isolates were investigated by the ability to produce antibacterial compounds using a semiquantitative modified well-diffusion assay (Castellano et al. 2010); *Lactobacillus plantarum* CRL691 (CERELA Culture Collection) and *Listeria innocua* 7 (from Unité de Recherches Latiéres et Genetique Appliqué, INRA, France) were used as indicator organisms. Biogenic amines formation was assayed using histidine and tyrosine as precursor amino acids according to Joosten and Northolt (1989). The strains were streaked on agar plates and incubated at 30°C for 2–5 days and color change from yellow to purple was considered as positive activity; *L. plantarum* CRL1485 (CERELA Culture Collection) was used as positive indicator organism. Antibiotic susceptibility test was performed applying the disk diffusion assay according to CLSI guidelines (CLSI 2006) using Mueller Hinton agar (Becton Dickinson, USA) and test disks for chloramphenicol (30 μg), erythromycin (15 μg) and tetracycline (30 μg). Sample preparations and analyses were performed in triplicate and two independents assays for each assay were carried out.

## Results and discussion

**Figure 1:**
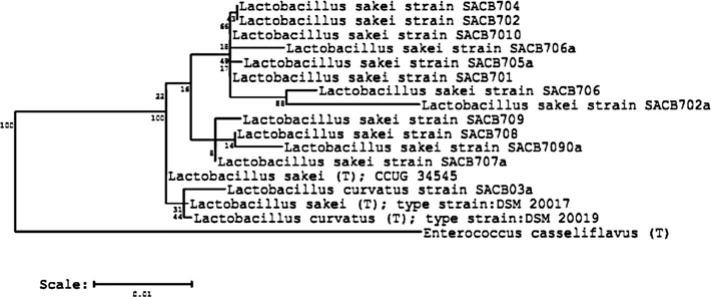
**Phylogenetic tree of 13 LAB strains isolated from fresh anchovy based on their 16S rRNA gene sequences.** The tree was constructed using the Ribosomal Database Project Release 10 (RDP) package (http://rdp.cme.msu.edu). *Enterococcus casseliflavus* was used as outgroup reference strain.

As previously reported by Belfiore et al. (2010), total bacterial viable count in fresh anchovies was 5.87 ± 0.19 log CFU/g, while LAB counts were 4.43 ± 1.67 log CFU/g. One hundred and twenty two isolates randomly picked from MRS agar plates were divided based on Gram reaction, morphology and catalase activity, into: i) Gram-positive, catalase-negative rods (15 isolates); ii) Gram-positive, catalase-negative cocci-bacilli (56 isolates); and iii) Gram-positive, catalase-positive cocci (50 isolates). Gram-positive, catalase-negative cocci-bacilli were identified by ribotyping as *Leuc. mesenteroides* and *Leuc. carnosum* (Belfiore et al. 2010). In the present study, the 15 Gram-positive, catalase-negative rod morphology isolates present in anchovies were identified and characterized phenotypically and technologically. Analysis of the partial (500–700 nt) 16S rDNA gene sequences of these isolates, blasted against the Genbank database, showed that all strains displayed a 97-99% similarity level with *L. sakei* and *L. curvatus*. The phylogenetic tree based on 13 of these sequences revealed close relationships among the isolates and other lactobacilli strains used as reference strains (Figure 1). The isolates SACB_7_05 and SACB_7_010a showed a 97% sequence similarity with *L. sakei/L. curvatus* strains; however, their nucleotide sequences were excluded since they were shorter than 380 nt. The occurrence of *L. sakei/L. curvatus* has also been previously reported in marinated fish products (Lyhs et al. 2002;Lyhs and Björkroth 2008). Even when the 16S and 23S rRNA genes have been widely used to define bacterial phylogenetic relationships (Fox et al. 1992), the diversity found in the 16S rRNA gene sequence of these two closely related species is insufficient for an accurate phylogenic distinction (Rachman et al. 2003;Najjari et al. 2008).

*L. sakei* and *L. curvatus* species are known to display a range of similar phenotypic traits that have resulted in difficulties for their differentiation (Samelis et al. 1995;Klein et al. 1996). These species only differ in the hydrolysis of arginine, fermentation of melibiose, xylose and haem-dependent catalase, among which atypical strains deviating from the standard patterns are often present (Berthier and Ehrlich 1999). In this study and based on those reported by these authors, 14 out of 15 isolates exhibited typical *L. sakei* phenotypic features: catalase activity, melibiose fermentation ability except that hydrolysis of arginine was absent in 4 of them (SACB_7_01, SACB_7_02a, SACB_7_06a and SACB707a). Only SACB03a which was catalase-, melibiose- and arginine-negative showed a typical *L. curvatus* profile (Table 2). Nevertheless, neither *L. sakei* nor *L. curvatus* fermented xylose as did *L. graminis* DSM20719 as previously reported by Berthier and Ehrlich (1999).

**Table 2:** **Phenotypic and technological characterization of LAB strains isolated from anchovies**

						Fermentation of		NaCl tolerance		
Strains	Antibacterial compounds against ***Listeria***	Biogenic amines	Antibiotic susceptibility	Catalase activity	Arginine hydrolysis	Melibiose	Xylose	10%	15%	18%
23K^a^	ND	ND	ND	+	+	+	-	ND	ND	ND
CRL978^b^	ND	ND	ND	+	ND	ND	-	ND	ND	ND
CRL705^c^	+	ND	ND	-	-	ND	-	ND	ND	ND
CRL1000^d^	ND	ND	ND	-	-	-	-	ND	ND	ND
DSM20719^T^	ND	ND	ND	ND	ND	-	+	ND	ND	ND
SACB03a	-	-	-	-	-	-	-	+*	+	+§
SACB_7_01	-	-	-	+	-	w	-	+	-	-
SACB_7_02	-	-	-	+	+	+	-	+	-	-
SACB_7_04	-	-	-	+	+	+	-	+**	+	+§
SACB_7_05	-	-	-	+	+	+	-	+	-	-
SACB_7_06	-	-	-	+	+	+	-	+	-	-
SACB_7_08	-	-	-	+	+	+	-	+	-	-
SACB_7_09	-	-	-	+	+	w	-	+	-	-
SACB_7_010	-	-	-	+	+	+	-	+	-	-
SACB_7_02a	-	-	-	+	-	+	-	+	-	-
SACB_7_05a	-	-	-	+	+	+	-	+	+	+
SACB_7_06a	-	-	-	+	-	w	-	+	-	-
SACB_7_07a	-	-	-	+	-	+	-	+	+	+
SACB_7_09a	-	-	-	+	+	w	-	+	+	+
SACB_7_010a	-	-	-	+	+	w	-	+	-	-

^a,b^*Lactobacillus sakei* reference strains; ^c,d^*Lactobacillus curvatus* reference strains; ^T^*Lactobacillus graminis* type strain; w: weak; ND: not determined; strains still viable at 120 (*), 240 (**) and 15 (§) days.

**Figure 2:**
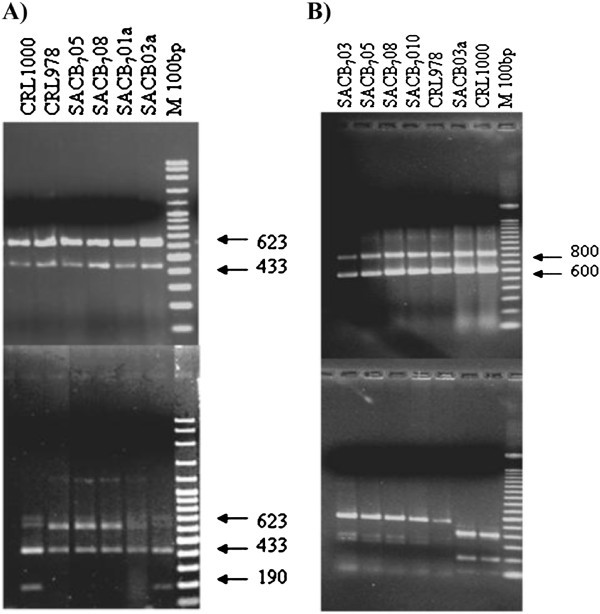
**Electrophoresis of*Hind*III-digested PCR products of the multiplex reaction (A) and*Taq*I digestion patterns (B) of*Lactobacillus*isolates SACB**_**7**_**01, SACB**_**7**_**01a, SACB**_**7**_**05, SACB**_**7**_**08, SACB03a.***Lactobacillus sakei* CRL978 and *Lactobacillus curvatus* CRL1000 were used as type strains; 100-bp DNA ladder was used as molecular weight marker.

To confirm these results, two molecular methods previously proposed to discriminate between *L. sakei* and *L. curvatus* were applied. When the multiplex PCR-REA method (Lee et al. 2004) was used, only the 623-bp amplified fragment from *L. curvatus* contained a single *Hin*dIII recognition site. As shown in Figure 2A, *Hin*dIII-restriction of the 623 bp PCR product into two fragments of 190 and 433 bp was only observed in the type strain *L. curvatus* CRL1000 and in the isolate SACB03a, whereas the 623 bp fragment from other anchovy isolates remained intact after enzyme treatment; this profile being characteristic of *L. sakei.* In the second method (Berthier and Ehrlich 1998), the species-specific primers 16S/p2 and 23S/p7 used were designed to amplify a DNA fragment containing the 16S-23S rDNA intergenic spacer region (ISR). RFLP of the PCR-amplified 16S-23S rDNA has proven to be a rapid method to characterize bacterial isolates and populations (Ruiz et al. 2000;Kabadjova et al. 2002;Rachman et al. 2003). The PCR product from *L. sakei, L. curvatus, L. graminis, L. paraplantarum, L. plantarum* and *L. pentosus* always consist of two ISR amplicons designated as small (S-ISR; 600 bp) and large (L-ISR; 800 bp) (Berthier and Ehrlich 1998). As shown in Figure 2B, PCR-amplified 16S-23S ISR of *L. curvatus* CRL1000, *L. sakei* CRL978 and anchovy isolates preliminary assigned as *L. sakei* (SACB_7_01, SACB_7_05, SACB_7_08 and SACB_7_010) and *L. curvatus* (SACB03a) yielded an identical pattern containing S-ISR and L-ISR distinct bands. RFLP with *Taq*I generated two characteristic profiles: four bands (600, 290, 330 and 100 bp) and three bands (400, 200 and 100 bp) profiles were generated for *L. sakei* CRL978 and *L. curvatus* CRL1000 reference strains, respectively. Again, isolate SACB03a displayed a *L. curvatus* RFLP characteristic pattern (Figure 2B), while the remaining isolates exhibited a *L. sakei* profile (data not shown). The applied methods proved to be reliable for anchovy isolates showing a strong agreement with phenotypic approaches in terms of species delineation, data suggesting that isolate SACBO3a may be identified as *L. curvatus*, while the other 14 isolates may be assigned as *L. sakei*. When strains biodiversity was assessed, the fingerprints of RAPD reactions using primers M13, RAPD1 and RAPD2 showed differences in the number of bands, fragment size and intensity suggesting a considerable polymorphism among the strains identified as *L. sakei* (data not shown). The observed intraspecies diversity is in coincidence with those reported by Chaillou et al. (2009) who revealed that many different *L. sakei* genotypes may be isolated from similar meat/fish products suggesting intraspecies genomic diversity may be required for successful adaptation.

From a technological standpoint and considering salt as a barrier to be overcome by LAB in anchovy-based products, the resistance of *L. sakei* isolates to NaCl was evaluated. Due to pelagic fish such as anchovies are excellent source of lipids, proteins, amino acids and vitamins and considering the high growth rate and biomass yield for LAB growing in a media supplemented with fish-based peptones (Horn et al. 2005), a slurry prepared from anchovy muscle added with glucose was used in this study to evaluate the growth ability of *L. sakei* strains in presence of NaCl (Table 2). Tolerance to 10% NaCl was observed for all isolates, *L. curvatus* SACB03a and *L. sakei* SACB704 being able to survive under this condition until 120 and 240 days, respectively. However, when tolerance to 15% and 18% NaCl was assayed, only four *L. sakei* strains (SACB_7_04, SACB_7_05a, SACB_7_07a, SACB_7_09a) and *L. curvatus* SACB03a were able to survive for 15 days in the presence of such high salt levels. Similar halotolerance was observed for *Lactococcus lactis* isolated from the gastrointestinal tract of coastal fish indicating that LAB are capable to adapt to continuously changing aquatic conditions such as periodic differences in osmotic pressure, dissolved oxygen and temperature (Itoi et al. 2009). Besides the lack of *L. curvatus* and *L. sakei* strains to produce dextran from sucrose, when safety traits were evaluated (Table 2), they were sensitive to chloramphenicol, tetracycline and erythromycin, suggesting they are free of potentially transferable resistance factors (EFSA 2007). In addition, no decarboxylation of tyrosine and histidine to produce biogenic amines was observed among anchovy strains and were unable to produce antibacterial compounds against the used indicator organisms.

Although many studies have described the natural and variable occurrence of LAB in fish (Ringø and Gatesoupe 1998;Ringø et al. 2002;Bucio et al. 2006;Michel et al. 2007), there are only a few studies reporting the presence of LAB in anchovies (*Engraulis anchoita*). *Pediococcus halophilus* (now classified as *Tetragenococcus halophilus*) has been described as the main species in salted anchovy by Villar et al. (1985) while more recently Najjari et al. (2008) reported the isolation of *L. sakei* from raw, salted and conserved in oils anchovies. In a previous study *Leuc. mesenteroides* and *Leuc. carnosum* were identified as part of LAB microbiota from fresh anchovies (Belfiore et al. 2010). Here, most of the identified lactobacilli were assigned to *L. sakei* species in coincidence with Najjari et al. (2008). Among *Lactobacillus* identified strains, a high ability to survive in saline environment must be highlighted for *L. sakei* SACB_7_04 and *L. curvatus* SACB03a. Since salt is often used as a hurdle to preserve fish and fish products, the high salt tolerance and the lack of negative safety traits exhibited by these strains may represent an advantage to be used as functional culture in salted anchovy-based products.

## References

[CR1] Balcázar JL, de Blas I, Ruiz-Zarzuela I, Vendrell D, Gironés O, Muzquiz J (2007). Sequencing of variable regions of the 16S rRNA gene for identification of lactic acid bacteria isolated from the intestinal microbiota of healthy salmonids. Comp Immunol Microbiol Infect Dis.

[CR2] Belfiore C, Björkroth J, Vihavainen E, Raya R, Vignolo G (2010). Characterization of *Leuconostoc* strains isolated from fresh anchovy (*Engraulis anchoita*). J Gen Appl Microbiol.

[CR3] Berthier F, Ehrlich SD (1998). Rapid species identification within two groups of closely related lactobacilli using PCR primers that target the 16S/23S rRNA spacer region. FEMS Microbiol Lett.

[CR4] Berthier F, Ehrlich SD (1999). Genetic diversity within *Lactobacillus sakei* and *Lactobacillus curvatus* and design of PCR primers for its detection using randomly amplified polymorphic DNA. Int J Syst Bacteriol.

[CR5] Bucio A, Hartemink R, Schrama JW, Verreth J, Rombouts FM (2006). Presence of lactobacilli in the intestinal content of freshwater fish from a river and from a farm with a recirculation system. Food Microbiol.

[CR6] Cabrer AI, Casales MR, Yeannes MI (2002). Physical and chemical changes in anchovy (*Engraulis anchoita*) flesh during marination. J Aquat Food Prod Technol.

[CR7] Castellano P, González C, Carduza F, Vignolo G (2010). Protective action of *Lactobacillus curvatus* CRL705 on vacuum-packaged raw beef. Effect on sensory and structural characteristics. Meat Sci.

[CR8] Chaillou S, Daty M, Baraige F, Dudez A, Anglade P, Jones R, Alpert CA, Champomier-Vergés M, Zagorec M (2009). Intraspecies genomic diversity and natural population structure of the meat-borne lactic acid bacterium *Lactobacillus sakei*. Appl Environ Microbiol.

[CR9] (2006). Approved Standard M2-A9. Performance standards for antimicrobial disk susceptibility tests.

[CR10] Cocconcelli PS, Porro D, Galandini S, Senini L (1995). Development of RAPD protocol for typing of strains of lactic acid bacteria and enterococci. Lett Appl Microbiol.

[CR11] Cole JR, Wang Q, Cardenas E, Fish J, Chai B, Farris RJ, Kulam-Syed-Mohideen AS, McGarrell DM, Marsh T, Garrity GM, Tiedje JM (2008). The Ribosomal Database Project: improved alignments and new tools for rRNA analysis. Nucleic Acids Res.

[CR12] de Man J, Rogosa M, Sharpe M (1960). A medium for the cultivation of lactobacilli. J Appl Bacteriol.

[CR13] Edwards U, Rogall T, Blocker H, Emde M, Böttger EC (1989). Isolation and direct complete nucleotide determination of entire genes. Characterization of a gene coding for 16S-ribosomal RNA. Nucleic Acids Res.

[CR14] European Food Safety Agency (EFSA) (2007). Introduction of a qualified presumption of safety (QPS) approach for assessment of selected microorganisms referred to EFSA. EFSA J.

[CR15] Fox GE, Wisotzkey JD, Jurtshuk P (1992). How close is close: 16S rRNA sequence identity may not be sufficient to guarantee species identity. Int J Syst Bacteriol.

[CR16] González-Rodríguez MN, Sanz J-J, Santos J-A, Otero A, García-López M-L (2002). Numbers and types of microorganisms in vacuum-packed cold-smoked freshwater fish at the retail level. Int J Food Microbiol.

[CR17] Gram L, Dalgaard P (2002). Fish spoilage bacteria – problems and solutions. Curr Opin Biotechnol.

[CR18] Gram L, Huss HH (1996). Microbiological spoilage of fish and fish products. Food Microbiol.

[CR19] Gürtler V, Stanisich V (1996). New approaches to typing and identification of bacteria using the 16S-23S rDNA spacer region. Microbiol.

[CR20] Hasan MR, Halwart M (2009). Fish and feed inputs for aquaculture. Practices, sustainability and implications.

[CR21] Horn SJ, Aspmo SI, Vü E (2005). Growth of *Lactobacillus plantarum* in media containing hydrolysates of fish viscera. J Appl Microbiol.

[CR22] Huey B, Hall J (1989). Hypervariable DNA fingerprinting in *Escherichia coli*: minisatellite probe from bacteriophage M13. J Bacteriol.

[CR23] Itoi S, Yuasa K, Washio S, Abe E, Ikuno E, Sugita H (2009). Phenotypic variation in *Lactococcus lactis* subsp. *lactis* isolates derived from intestinal tracts of marine and freshwater fish. J Appl Microbiol.

[CR24] Joosten HL, Northolt MD (1989). Detection, growth and amine-producing capacity of lactobacilli in cheese. Appl Environ Microbiol.

[CR25] Kabadjova P, Dousset X, Le Cam V, Prevost H (2002). Differentiation of closely related *Carnobacterium* food isolates based on 16S-23S ribosomal DNA intergenic spacer region polymorphism. Appl Environ Microbiol.

[CR26] Klein G, Dicks LM, Pack A, Zimmermann K, Dellaglio F, Reuter G (1996). Emended descriptions of *Lactobacillus sake* (Katagiri, Ktahara, and Fukami) and *Lactobacillus curvatus* (Abo-Elnaga and Kandler): numerical classification revealed by protein fingerprinting and identification based on biochemical patterns and DNA-DNA hybridizations. Int J Syst Bacteriol.

[CR27] Kopermsub P, Yunchalard S (2010). Identification of lactic acid bacteria associated with the production of plaa-som, a traditional fermented fish product of Thailand. Int J Food Microbiol.

[CR28] Kullen MJ, Sanozky-Dawes RB, Crowell DC, Klaenhammer TR (2000). Use of the DNA sequence of variable regions of the 16S rRNA gene for rapid and accurate identification of bacteria in the *Lactobacillus acidophilus* complex. J Appl Microbiol.

[CR29] Lane DJ, Pace B, Olsen GJ, Stahl DA, Sogin BM, Pace NR (1985). Rapid determination of 16S ribosomal RNA sequences for phylogenetic analyses. Proc Natl Acad Sci USA.

[CR30] Lauzon HL, Ringø E, Lahtinen S, Ouwehand A, Salminen S, Von Wright A (2012). Prevalence and application of lactic acid bacteria in aquatic environments. Lactic Acid Bacteria. Microbiological and functional aspects.

[CR31] Lee J, Jang J, Kim B, Jeong G, Han H (2004). Identification of *Lactobacillus sakei* and *Lactobacillus curvatus* by multiplex PCR-based restriction enzyme analysis. J Microbiol Methods.

[CR32] Leroi F (2010). Occurrence and role of lactic acid bacteria in seafood. Food Microbiol.

[CR33] Liu JY, Li AH, Ji C, Yang WM (2009). First description of a novel *Weissella* species as an opportunistic pathogen for rainbow trout *Oncorhynchus mykiss* (Walbaum) in China. Vet Microbiol.

[CR34] Lyhs U, Björkroth J (2008). *Lactobacillus sakei*/*curvatus* is the prevailing lactic acid bacterium group in spoiled maatjes herring. Food Microbiol.

[CR35] Lyhs U, Korkeala H, Björkroth J (2002). Identification of lactic acid bacteria from spoiled, vacuum-packaged “gravad” rainbow trout using ribotyping. Int J Food Microbiol.

[CR36] Matamoros S, Pilet MF, Gigout F, Prévost H, Leroi F (2009). Selection and evaluation of seafood-borne psychrotrophic lactic acid bacteria as inhibitors of pathogenic and spoilage bacteria. Food Microbiol.

[CR37] McClesky CS, Faville LW, Barnett Rex O (1947). Characteristics of *Leuconostoc mesenteroides* from cane juice. J Bacteriol.

[CR38] Michel C, Pelletier C, Boussaha M, Douet D-G, Lautraite A, Taillez P (2007). Diversity of lactic acid bacteria associated with fish and the fish farm environment, established by amplified rRNA gene restriction analysis. Appl Environ Mirobiol.

[CR39] (2008). Desembarques de capturas marítimas totales.

[CR40] Najjari A, Ouzari H, Boudanous A, Zagorec M (2008). Method for reliable isolation of *Lactobacillus sakei* strains originating from Tunisian seafood and meat products. Int J Food Microbiol.

[CR41] Pospiech A, Neumann B (1995). A versatile quick-prep of genomic DNA from Gram-positive bacteria. Trends Genet.

[CR42] Rachman C, Kabadjova P, Prévost H, Dousset X (2003). Identification of *Lactobacillus alimentarius* and *Lactobacillus farciminis* with 16S-23S rDNA intergenic spacer region polymorphism and PCR amplification using species-specific oligonucleotide. J Appl Microbiol.

[CR43] Ringø E, Gatesoupe FJ (1998). Lactic acid bacteria in fish: a review. Aquaculture.

[CR44] Ringø E, Wesmajervi MS, Bendiksen HR, Berg A, Olsen RE, Johnsen T, Mikkelsen H, Seppola M, Strom E, Holzapfel W (2002). Identification and characterization of *Carnobacterium* isolated from fish intestine. Syst Appl Microbiol.

[CR45] Ruiz A, Poblet M, Mas A, Guillamón JM (2000). Identification of acetic acid bacteria by RFLP of PCR-amplified 16S rDNA and 16S-23S rDNA intergenic spacer. Int J Evol Microbiol.

[CR46] Ruiz-Zarzuela I, de Blas I, Gironés O, Ghittino C, Muzquiz JL (2005). Isolation of *Vagococcus salmoninarum* in rainbow trout *Oncorhynchus mykiis* (Walbaum), Broodstocks: characterization of the pathogen. Vet Res Com.

[CR47] Sambrook L, Frits EF, Maniatis T (1989). Molecular Cloning: A Laboratory Manual.

[CR48] Samelis J, Tsakalidou E, Metaxopoulos J, Kalantzopoulos G (1995). Differentiation of *Lactobacillus sakei* and *Lactobacillus curvatus* isolated from naturally fermented dry salami. J Appl Bacteriol.

[CR49] Seppola M, Olsen ER, Sandaker E, Kanapathippillai P, Holzapfel W, Ringǿ E (2006). Random amplification of polymorphic DNA (RAPD) typing of carnobacteria isolated from hindgut chamber and large intestine of Atlantic cod (*Gadus morhua I.*). Syst Appl Microbiol.

[CR50] Svanevik CS, Lunestad BT (2011). Characterization of the microbiota of Atlantic mackerel (*Scomber scombrus*). Int J Food Microbiol.

[CR51] Thapa N, Pal J, Tamang JP (2006). Phenotypic identification and technological properties of lactic acid bacteria isolated from traditionally processed fish products of the Eastern Himalayas. Int J Food Microbiol.

[CR52] Vendrell D, Balcázar JL, Ruiz-Zarzuela I, de Blas I, Gironés O, Múzquiz JL (2006). *Lactococcus garvieae* in fish: a review. Comp Immunol Microbiol Infect Dis.

[CR53] Villar M, de Ruiz P, Holgado A, Sanchez JJ, Trucco R, Oliver G (1985). Isolation and characterization of *Pediococcus halophilus* from salted anchovies (*Engraulis anchoita*). Appl Environ Microbiol.

